# In Vitro Wear of a Novel Vitamin E Crosslinked Polyethylene Lumbar Total Joint Replacement

**DOI:** 10.3390/bioengineering10101198

**Published:** 2023-10-15

**Authors:** Ryan L. Siskey, Ronald V. Yarbrough, Hannah Spece, Scott D. Hodges, Steven C. Humphreys, Steven M. Kurtz

**Affiliations:** 1 Exponent Inc., Philadelphia, PA 19104, USA; 2 3Spine, Chattanooga, TN 37402, USA; 3 School of Biomedical Engineering, Science, and Health Systems, Drexel University, Philadelphia, PA 19104, USA

**Keywords:** lumbar spine, total joint replacement, vitamin E, antioxidant, highly crosslinked polyethylene (HXLPE), wear, impingement, abrasion, preclinical testing, design

## Abstract

Background: A novel, lumbar total joint replacement (TJR) design has been developed to treat degeneration across all three columns of the lumbar spine (anterior, middle, and posterior columns). Thus far, there has been no in vitro studies that establish the preclinical safety profile of the vitamin E-stabilized highly crosslinked polyethylene (VE-HXLPE) lumbar TJR relative to historical lumbar anterior disc replacement for the known risks of wear and impingement faced by all motion preserving designs for the lumbar spine. Questions/Purpose: In this study we asked, (1) what is the wear performance of the VE-HXLPE lumbar TJR under ideal, clean conditions? (2) Is the wear performance of VE-HXLPE in lumbar TJR sensitive to more aggressive, abrasive conditions? (3) How does the VE-HXLPE lumbar TJR perform under impingement conditions? Method: A lumbar TJR with bilateral VE-HXLPE superior bearings and CoCr inferior bearings was evaluated under clean, impingement, and abrasive conditions. Clean and abrasive testing were guided by ISO 18192-1 and impingement was assessed as per ASTM F3295. For abrasive testing, CoCr components were scratched to simulate in vivo abrasion. The devices were tested for 10 million cycles (MC) under clean conditions, 5 MC under abrasion, and 1 MC under impingement. Result: Wear rates under clean and abrasive conditions were 1.2 ± 0.5 and 1.1 ± 0.6 mg/MC, respectively. The VE-HXLPE components demonstrated evidence of burnishing and multidirectional microscratching consistent with microabrasive conditions with the cobalt chromium spherical counterfaces. Under impingement, the wear rates ranged between 1.7 ± 1.1 (smallest size) and 3.9 ± 1.1 mg/MC (largest size). No functional or mechanical failure was observed across any of the wear modes. Conclusions: Overall, we found that that a VE-HXLPE-on-CoCr lumbar total joint replacement design met or exceeded the benchmarks established by traditional anterior disc replacements, with wear rates previously reported in the literature ranging between 1 and 15 mg/MC. Clinical Relevance: The potential clinical benefits of this novel TJR design, which avoids long-term facet complications through facet removal with a posterior approach, were found to be balanced by the in vitro tribological performance of the VE-HXLPE bearings. Our encouraging in vitro findings have supported initiating an FDA-regulated clinical trial for the design which is currently under way.

## 1. Introduction

Traditional lumbar anterior disc replacement (ADR) was developed as an alternative to treatment with fusion for select patients with degenerative disc disease [1,2,3]. By preserving the motion of the diseased level, the hope was that lumbar ADR would avert adjacent segment degeneration and potentially prevent adjacent segment disease [1,3]. Several different lumbar ADR designs have been clinically introduced in the United States since the early 2000s, most with conventional ultra-high molecular weight polyethylene (UHMWPE) bearings articulating against a cobalt chromium (CoCr) alloy counterface. In strict FDA monitored clinical trials, lumbar ADRs have demonstrated improved outcomes over fusion at intermediate- and long-term follow-up [4]. However, despite compelling scientific evidence supporting the clinical benefits of motion preservation as an alternative to fusion [5], the technology has limitations that have diminished its acceptance and utilization by surgeons [6].

By design, lumbar ADRs are focused on addressing discogenic back pain, and careful screening is needed to rule out facet degeneration and other potential sources of pain generation from the posterior column. The anterior approach for installing a lumbar ADR is more challenging than other surgical approaches to the lumbar spine, especially for revision ADR surgery [7]. Furthermore, many of the lumbar ADRs approved for use in the United States today employ polyethylene biomaterials that were state-of-the-art for hip and knee joints back in the 1990s but are known to be susceptible to long-term in vivo oxidation, wear, and fatigue damage [3,8]. Because these in vivo material changes are influenced by unalterable factors (e.g., patient anatomy and biology, device characteristics and application) years of effort were dedicated to improving the quality and wear resistance of the polyethylene biomaterials themselves [9,10,11]. Now, contemporary hip and knee joints predominantly use either highly crosslinked biomaterials that are more resistant to wear, or highly crosslinked and antioxidant-stabilized polyethylene to also limit in vivo oxidation [12,13].

A novel total joint replacement (TJR) design has been developed that treats degeneration across all three columns of the lumbar spine (anterior, middle, and posterior) [14,15]. Instead of relying on a single bearing inserted via an anterior surgical approach, the lumbar TJR consists of two bilateral bearings, inserted using the same posterior approach as a posterior lumbar interbody fusion [14]. Biomechanically, the dual bilateral bearing design allows for flexion and extension for sagittal balance but constrains lateral bending and axial rotation to provide stability [15]. Thus, unlike existing disc replacements, the TJR is a replacement for the function of both the disc and the facets [14,15]. Cadaveric biomechanical studies of the TJR design by Padwardhan and colleagues [15] have verified that it affords a physiological range and quality of motion to the lumbar spine at treated and adjacent levels during sitting to standing. Initial TJR designs incorporated bilateral metal-on-metal bearings because of their durability and the limitations of polyethylene at the time, with the tradeoff of potential metal ion release and adverse local tissue reactions [14]. Concerns about potential metal particulate release across orthopedic devices [16] prompted the updating of the TJR design to incorporate bearings of state-of-the-art vitamin E-stabilized, highly crosslinked polyethylene (VE-HXLPE) [17,18]. Although VE-HXLPE has a successful clinical history as a wear-, fatigue-, and oxidation-resistant bearing material in large joint (hip and knee) total joint replacements [17,19], its long-term clinical performance for lumbar TJR remains to be determined.

Prior to evaluating VE-HXLPEs clinically in lumbar TJR, it was essential to evaluate the design for its durability by wear and impingement testing, which have been shown to be clinically relevant, potential failure modes for lumbar ADRs [20]. Over the past two decades, international standards for wear and impingement testing have been developed for lumbar ADRs [21,22], serving as a benchmark for investigational lumbar TJR designs. Specifically, we addressed three research questions: (1) What is the wear performance of the VE-HXLPE lumbar TJR under ideal, clean conditions? (2) Is the wear performance of the VE-HXLPE in lumbar TJR sensitive to more aggressive, abrasive conditions? (3) How does the VE-HXLPE lumbar TJR perform under impingement conditions? Collectively, these in vitro studies serve to establish the preclinical safety profile of the VE-HXLPE lumbar TJR relative to historical lumbar ADR for the known risks of wear and impingement faced by all motion-preserving designs for the lumbar spine.

## 2. Methods

**Figure 1:**
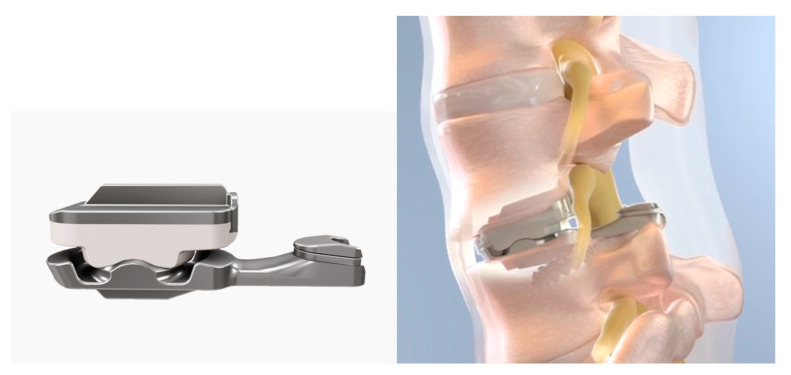
Bilateral VE-HXLPE lumbar TJR design evaluated in wear and impingement testing.

A total joint replacement (MOTUS^®^: 3Spine, Chattanooga, TN, USA) with bilateral VE-HXLPE superior articulating sockets and CoCr inferior spherical surfaces was evaluated under clean, impingement, and abrasive testing conditions. The design of the VE-HXLPE lumbar TJR (Figure 1) incorporated the bearing surface design of an earlier generation lumbar TJR [14,15] with its foundational biomechanical and clinical performance. The anterior and posterior aspects of the VE-HXLPE components were designed to accommodate potential impingement with the inferior CoCr component beyond the range of physiological motion.

### 2.1. Standard Wear Testing

ISO 18192-1 was used to guide the wear testing of coupons representative of the articulating surfaces of the lumbar TJR design shown in Figure 1 [22]. Specifically, six-degree-of-freedom spine wear simulators (MTS, Eden Prairie, MN, USA) were used for testing. The test consisted of six wear stations, two load soak stations, and two passive soak stations. Test and load soak control specimens were soaked in distilled water at room temperature for 48 h prior to testing to correct for fluid uptake by the superior component.

**Figure 2:**
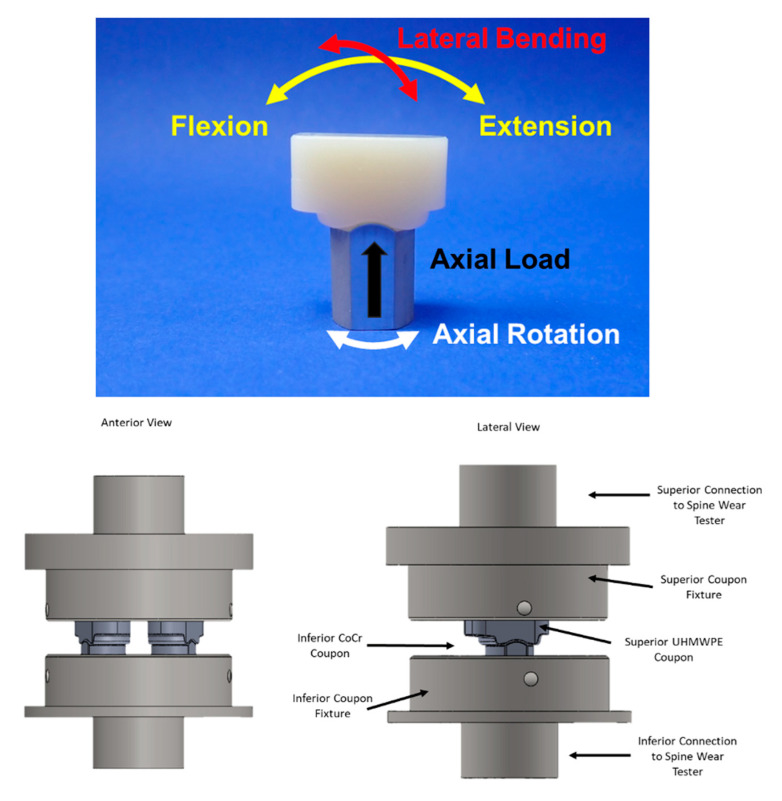
(**Top**) Image of one half of the lumbar TJR showing the assembly of the superior and inferior components and the applied motions and loading and (**bottom**) model of the fixture assembly showing the inferior and superior components clamped within the fixtures. This setup was used for both standard and abrasive wear testing.

Each device was assembled in the spine wear simulator using custom fixtures designed such that the center of rotation of the wear simulator’s flexion–extension and lateral bending axes matched the center of rotation of the device and was subjected to the loads and motions prescribed for lumbar disc prostheses in ISO 18192-1 (Figure 2). A motion profile with a constant frequency of 1.0 Hz was applied to each specimen, except for axial loading which occurred at a frequency of 2.0 Hz. The coupled axes were maintained with a constant sinusoidal amplitude control and included ±4.5° flexion–extension, ±2° lateral bending, ±2° rotation, and 600–2000 N compressive axial load. The phasing of the applied motions was consistent with the recommendations in ISO 18192-1. The symmetric ±4.5° flexion–extension angle is equivalent in total magnitude to the ISO recommended +6/−3° flexion–extension angle. Pilot testing was performed to ensure that the modified input would result in equal challenges to the bilateral articulating surfaces of the bearing design. Two devices were tested as active soak control specimens and were subjected to compressive axial load only, while two others were passively soaked to compensate for fluid uptake. The remaining samples were tested using the motion profile described above.

All samples remained lubricated at room temperature (23 ± 2 °C) in HyClone Wear Test Fluid (Bovine Serum), diluted to a protein concentration of 5 g/L, which also contained both an antibacterial and antifungal additive to prevent contamination due to bacteria and fungi. The test medium was changed every 0.5 million cycles (MC) to perform gravimetric analysis.

### 2.2. Adverse Abrasive Testing

**Figure 3:**
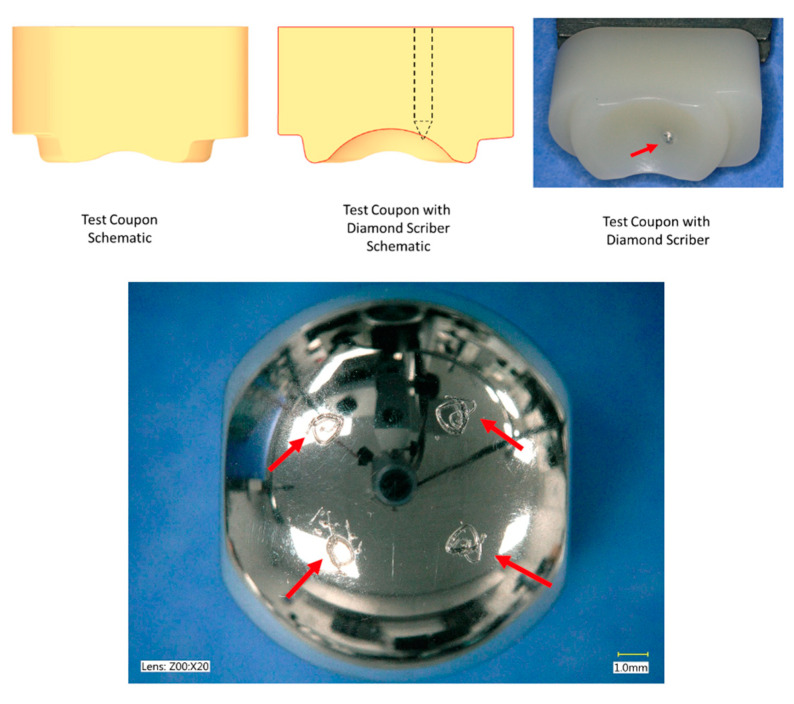
(**Top**) The modified superior test coupon with diamond scriber used to scratch the inferior components. The right image with red arrow shows the diamond scriber tip protruding through the UHMWPE component’s articulating surface. (**Bottom**) Representative image of an inferior component post-scratching procedure. The red arrows show the four scratches imparted in the quadrants of the articulating surface.

Abrasive testing was conducted using the identical fixtures, simulator, and loading and motion conditions as the standard tests described previously, with the exception that the CoCr counterfaces were intentionally scratched to mimic an adverse in vivo abrasive damage mode, consistent with third-body particulates entering the articulation. Prior to wear testing, a procedure was performed to scratch the CoCr components of the lumbar TJR design. For the scratching procedure, a custom fixture with a superior test coupon modified to hold a diamond scribe (Product Number 1984A17, McMaster-Carr, Elmhurst, IL, USA) was used to create scratch patterns on each of the four quadrants of each inferior component (Figure 3). The ISO lumbar profile was run to create an elliptical scratch pattern on each of the four quadrants of the CoCr components. White light interferometry measurements confirmed that the depth of each elliptical scratch pattern was approximately 10 microns on each of the four quadrants of the inferior CoCr components for the test. After severe scratching was induced on the CoCr components, wear tests were run paired against initially pristine, unworn VE-HXLPE components, in accordance with ISO 18192-1, as described in the previous section, up to 5 MC.

### 2.3. Impingement Testing

Impingement was modeled and assessed for 1 MC in accordance with ASTM F3295 [21]. Solid modeling was conducted using SolidWorks (Dassault Systèmes, Waltham, MA, USA) to develop an impingement protocol for the VE-HXLPE lumbar TJR for both testing size devices. The resulting analysis dictated that using an 8° initial impingement angle would be most representative of clinical, worst-case impingement behavior. The aligned initial impingement angle was validated experimentally using a servo hydraulic rotary actuator. During impingement testing, implant surfaces outside of the intended bearing surface were brought into cyclic contact to induce wear. In contrast to the standard and abrasive wear testing, which were conducted on test coupons representative of the polymer and metal bearing surfaces to facilitate precise gravimetric evaluation, impingement testing was conducted on eight sets of pristine, final-form superior and inferior lumbar TJR components. Both long and short component sizes were evaluated, because the smaller size represented a worst-case contact stress condition, while the largest size represents the worst-case contact area for the design under the modeled impingement conditions.

**Figure 4:**
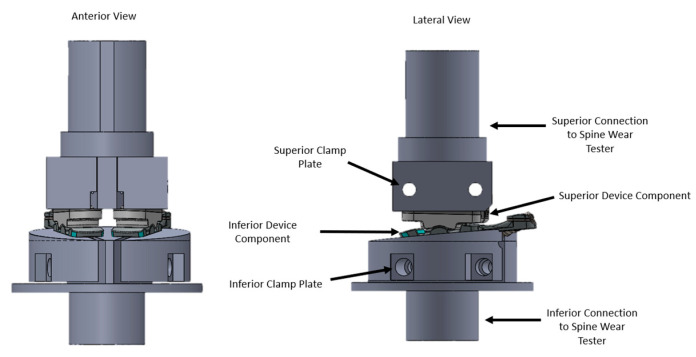
Model of the fixture assembly showing the inferior and superior components clamped within the fixtures.

Based on the results of modeling, experimental validation, and the limitations of the tester, an initial impingement angle of 8° was built into the interior fixture and used as the initial impingement input for testing of long and short implant sizes. Overall, the impingement conditions included a ±2° axial rotation, 1200 N static compressive axial load, and flexion/extension range of +/−4° centered at an 8° posterior angle (flexion range of 4–12°). The impingement testing utilized the same MTS simulators and lubricant conditions as the wear tests described in the preceding sections and fixtures as shown in Figure 4.

### 2.4. Interval Analyses

Wear and impingement tests were stopped every 0.25 MC up to 1.0 MC, and every 0.5 MC thereafter. Components were removed, cleaned, and desiccated as per the recommendations in ASTM F1714 as referenced in ASTM F2423 Components were photographed, and for gravimetric analysis the mass of each component was measured three times in rotation using a Sartorius CPA225D balance (Bohemia, New York, USA). The mass loss for each superior component was corrected for fluid absorption at the end of each cycle interval during testing. Specifically, the maximum increase in mass of the load soak and soak components was subtracted from each of the wear-tested sample masses at each interval due to the fact that the load soak samples demonstrated the most water absorption. The final material removal rate was calculated by fitting a regression line to the interval mass loss for each component. The individual wear rates were then averaged to obtain a representative wear rate for the device. The volumetric material removal rate was calculated by dividing final material removal rate with a nominal UHMWPE density of 0.935 g/cm^3^ for the superior components.

MicroCT was used to further characterize the penetration of the superior polyethylene components using a μCT 80 (Scanco Medical AG, Wangen-Brüttisellen, Switzerland) at a maximum voxel resolution of approximately 18 μm. A custom MatLab (MathWorks, Natick, MA, USA) code was written in order to calculate the dimensional changes for each device. In order to calculate the maximum dimensional changes, the surfaces of each device at the end of the test were automatically aligned with a surface generated from the sample at the beginning of the test [23]. This analysis results in a penetration map that can be used to visualize the combination of wear and deformation across the entire surface of the components.

## 3. Results

### 3.1. Standard Wear Testing

**Figure 5:**
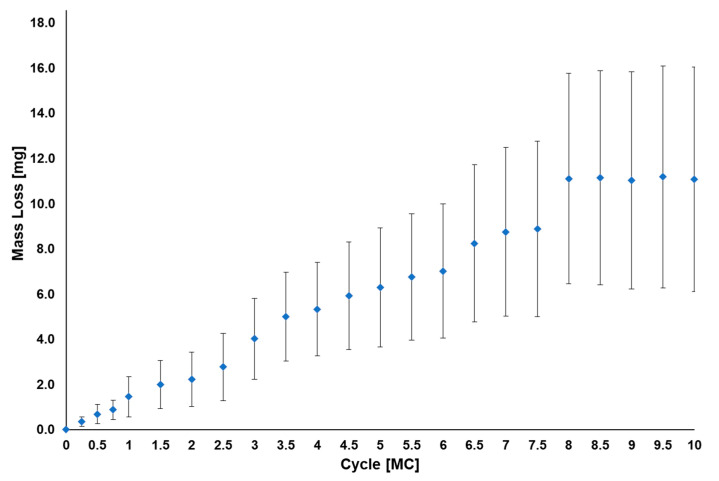
Average total mass loss for the VE-HXLPE components corrected for soaking (*n* = 6) during the standard clean wear test. Error bars represent one standard deviation.

**Figure 6:**
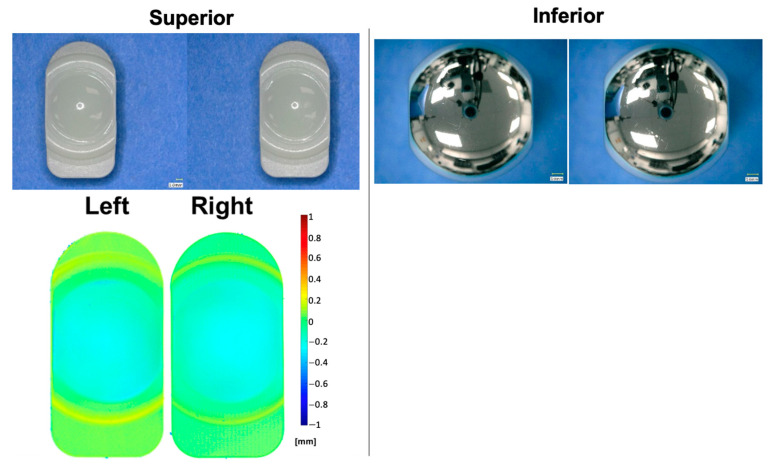
Representative micrographs (**top**) and penetration maps (**bottom**) of the left and right polyethylene and CoCr bearings after 10.0 MC during the standard clean wear test.

The VE-HXLPE wear rate during the standard test was 1.3 ± 0.5 mg/MC between 0 and 4 MC, and 1.1 ± 0.6 mg/MC between 4 and 10 MC, consistent with a stable, overall wear rate of 1.2 ± 0.5 mg/MC (Figure 5). The articulating surfaces of the VE-HXLPE superior components demonstrated a loss of machine marks consistent with microabrasive wear in the articulating surface. The worn regions of the VE-HXLPE components showed evidence of burnishing and multidirectional scratching and the CoCr inferior components demonstrated multidirectional scratching (Figure 6). No device components demonstrated evidence of mechanical or functional failure after 10.0 MC of testing. The penetration rate for the superior VE-HXLPE components was found to be 0.18 ± 0.04 mm/MC from 0 to 1. MC and then decreased to 0.01 ± 0.00 mm/MC from 1 to 10 MC. Generally, the penetration was observed to follow a similar trend to the mass loss measured for each component. Outward deformation of the bearing was also noted around the periphery of the component consistent with the stresses in the bearing when loaded with the ISO 18192-1 duty cycle.

### 3.2. Adverse Abrasive Testing

**Figure 7:**
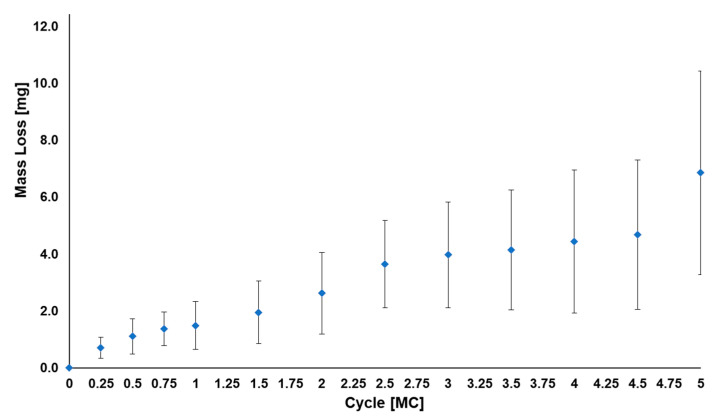
Average total mass loss for the VE-HXLPE components corrected for soaking (*n* = 6) during the adverse abrasive wear test. Error bars represent one standard deviation.

**Figure 8:**
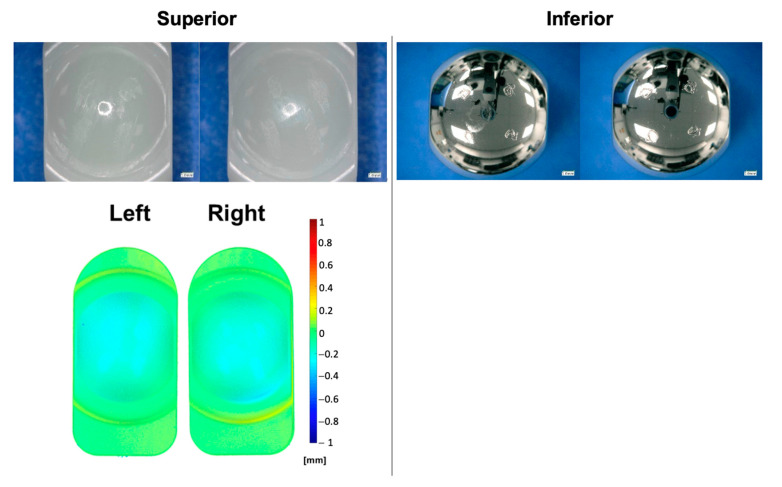
Representative micrographs (**top**) and penetration maps (**bottom**) of the left and right polyethylene and CoCr bearings after 5.0 MC during the adverse abrasive wear test.

The calculated average mass wear rate up to 5.0 MC was 1.1 ± 0.6 mg/MC for the superior VE-HXLPE components (Figure 7). After 5.0 MC, the bearing surfaces demonstrated evidence of abrasive wear at the articulating surface of the superior components coincident with intentionally imparted scratches on the inferior components (Figure 8). In the regions adjacent to the scratches, the machine marks of the superior articulating surface were worn away by 1.0 MC and the surface maintained a burnished appearance for the remainder of the test as anticipated. Despite the abrasive environment, the device components did not demonstrate evidence of mechanical or functional failure after 5.0 MC of testing. The CoCr inferior articulating surface was scratched intentionally at the start of the test and those scratches remained similar in morphology and magnitude for the duration of the test. The penetration rate for the superior components was found to be 0.26 ± 0.06 mm/MC from 0 to 1.0 MC and then decreased to 0.02 ± 0.01 mm/MC from 1 to 5 MC. Generally, the penetration was observed to follow a similar trend to the mass loss measured for each component. Outward deformation of the bearing was also noted around the periphery of the component which is consistent with the stresses in the bearing when loaded with the ISO 18192-1 duty cycle.

### 3.3. Impingement Testing

**Figure 9:**
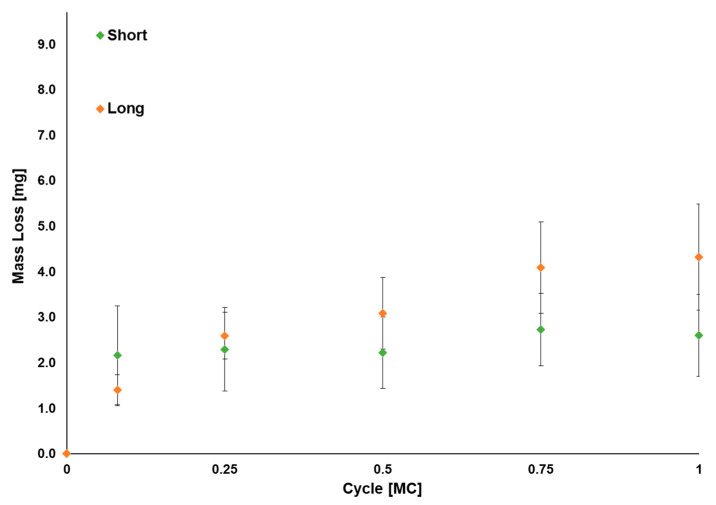
Average total mass loss for the VE-HXLPE components corrected for soaking (*n* = 6) during the impingement test. Error bars represent one standard deviation.

**Figure 10:**
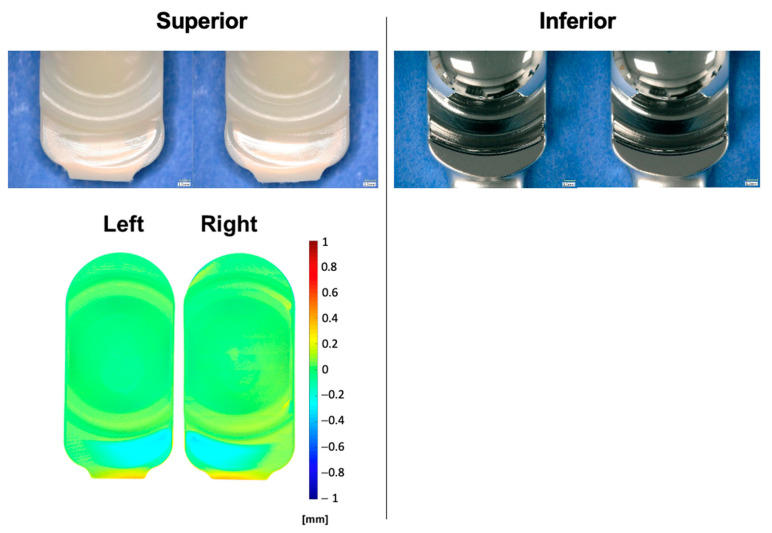
Representative micrographs (**top**) and penetration maps (**bottom**) of the left and right polyethylene and CoCr bearings after 1.0 MC during the impingement test.

The average mass wear rate of the VE-HXLPE components up to 1.0 MC for short and long implant sizes was 1.7 ± 1.1 mg/MC and 3.9 ± 1.1 mg/MC, respectively (Figure 9). The impingement regions of the VE-HXLPE components showed evidence of burnishing, deformation, and multidirectional scratching and the impingement regions of the CoCr inferior components demonstrated multidirectional scratching (Figure 10). These areas were in good agreement with the regions of impingement contact predicted by computational modeling. No device components demonstrated evidence of mechanical or functional failure after 1.0 MC of testing. Additionally, the screws and inferior endplates were inspected and did not show evidence of fretting or corrosion. The superior short VE-HXLPE components demonstrated an average penetration of 0.29 ± 0.07 mm for the wear stations and 0.13 mm for the load soak station across the 1.0 MC test. The superior long VE-HXLPE components demonstrated an average penetration of 0.17 ± 0.03 mm for wear stations and 0.03 for the load soak station across the 1.0 MC test.

## 4. Discussion

Wear and osteolysis continue to be important clinical concerns in both large joint and spinal arthroplasty [13,16,24,25]. In this study, we performed in vitro wear testing of a novel lumbar total joint replacement design that replicates the biomechanics and kinematics of the intervertebral disc and facet joint complex. Over the past two decades, international consensus standards have been developed by the spine arthroplasty community to assess the expected risks due to wear under clean, abrasive, and impingement conditions and to compare wear performance across different ADR designs. These standards were validated based on retrieval evidence of both mobile-bearing and fixed-bearing ADR designs that incorporate CoCr endplates articulating against a conventional UHMWPE insert [26,27]. We employed these established test methods, as well as an abrasive wear protocol, to evaluate a novel VE-HXLPE-on-CoCr lumbar TJR bearing design and found low wear rates under clean and adverse conditions.

Our study has limitations. In our in vitro simulations, we considered impingement and abrasive wear conditions as the most important adverse wear risks to address with bench testing, based on the clinical history and retrieval evidence from previous disc arthroplasty designs. Although our bench testing did not accommodate variations in surgical alignment or device positioning, we explored these potential risks using finite element modeling prior to undertaking the first physical bench tests to help ensure that the lumbar TJR design would be as forgiving as possible under misaligned and impingement test scenarios. More advanced finite element modeling with an anatomic spine model may be useful to confirm the tolerance of the LTJR design to surgical misalignment in the future. Additionally, in vitro studies are inherently limited in their ability to recreate clinical conditions, and the impact that some patient and surgical factors may have on device performance remain to be seen. The effect that more substantial counterface scratching from device damage or third body debris may have on abrasive wear is similarly undeterminable. Future studies involving short- and long-term clinical performance will be necessary once the data become available. Finally, we did not have a control group for comparison in our wear testing. However, the previous study results outlined in the following tables provide comparative values from wear tests of clinically relevant ADRs, and we used similar methods to those reported.

Wear and in vivo oxidation emerged as concerns for preserving the long-term desirable properties of polyethylene in the late 1990s, and many efforts were made to control and optimize processing without negatively impacting the mechanical and wear properties [28,29]. The first generation of HXLPEs were stabilized against in vivo oxidation by thermal processing, such as annealing or remelting, but these methods compromise the mechanical properties of the material [30]. More recently, antioxidants such as alpha tocopherol (vitamin E) have been introduced to polyethylene to protect the material against oxidation during processing, shelf storage, and in vivo use [17]. VE-HXLPE is a second-generation HXLPE material, which was clinically introduced in hip arthroplasty after 2005 [30]. Now, with over 15 years of clinical use in large-joint arthroplasty, VE-HXLPE has an established successful history in both total hip and knee arthroplasty, as summarized in recent systematic reviews [17,19]. The clinical track record of VE-HXLPE motivated the developers of lumbar TJRs to incorporate this bearing material into the design.

Our results for VE-HXLPE-on-CoCr LTJR wear under standard (clean) conditions met or exceeded the benchmarks established by traditional ADRs as published in the FDA’s Summary of Safety and Effectiveness Data (SSED) reports (Table 1) and in the clinical literature (Table 2). For comparison, mass-based wear rates from previous studies that used the same ISO 18192 testing standard ranged from 2.7 mg/MC to 13.8 mg/MC [27,31]. Our clean wear results (1.2 ± 0.5 mg/MC) were lower than the previously reported values, a finding that is particularly notable given the dual implant design of the lumbar TJR. The results from our adverse abrasive testing (1.1 ± 0.6 mg/MC) were similar to those of the clean wear results, suggesting that, although localized abrasion corresponding to the scratched areas of the CoCr counterface was present, the VE-HXLPE surface is robust against abrasive wear, similar to previous in vitro findings for the spine and large joints [32,33].

We also found that the VE-HXLPE construct showed similar wear under impingement compared to published data (Table 1 and Table 3). It is interesting to note that the development of standardized wear and impingement test methods for ADR evolved relatively recently in the mid-2000s with the clinical introduction of the first lumbar disc design in the United States (CHARITÉ, Depuy Synthes, West Chester, PA, USA) [26,27]. Although it has been superseded by other ADRs, as the trailblazer for disc arthroplasty, CHARITÉ was one of the most widely studied designs due in part to the availability of clinical retrievals [26,27] which enabled the validation of ADR wear and impingement test methods. However, it was also learned that an unconstrained design, such as CHARITÉ, could not be tested in precisely the same wear and impingement protocols as a more constrained, ball-in-socket design. For our research, we adopted the same, generally accepted, wear and impingement test methods as previous lumbar ADR designs (Table 1 and Table 3), which is a strength of the study. Overall, our measured wear rates were low and comparable with previous results.

Our findings are unsurprising when considering the history of polyethylene advancements for large joint arthroplasty. Wear and impingement are system properties, not material properties, and consequently are dependent on implant design, bearing materials, and testing conditions. Our understanding of all three of these factors has changed over time in large joint orthopedics, but remain in comparatively early stages of evolution in the field of spine arthroplasty [16]. Innovation in understanding of polyethylene wear mechanisms from in vitro wear testing, including the understanding of how crossing shear motion of the hip joint was responsible for elevated wear rates [42,43,44], led to the development of HXLPE which has been widely credited to have markedly reduced the risk of osteolysis and aseptic loosening in modern joint replacements [12,45]. Analysis of lumbar ADRs and the availability of retrieved total discs confirmed that similar wear mechanisms were also relevant to the spine [26,27]. Nevertheless, contemporary, lumbar ADR devices currently approved by the FDA employ conventional—not highly crosslinked—formulations of polyethylene in their designs. Additionally, in response to concerns about in vivo oxidation and the long-term preservation of desirable polyethylene properties [28,29,30], a second-generation of HXLPEs incorporating antioxidants were clinically introduced in total hip arthroplasty [30]. Now with over 15 years of clinical use in large joint arthroplasty, VE-HXLPE has an established history of clinical success for both hip and knee devices [17,19].

The track record of VE-HXLPE motivated the developers of lumbar TJR to incorporate this second-generation polyethylene bearing material into their design. Such design decisions are of utmost importance given the inherent risk of impingement and wear in motion-preserving lumbar spine devices. Previous studies have shown that, like large joint arthroplasty devices, lumbar ADR devices may produce wear debris capable of inducing inflammatory reactions [20]. Device impingement may further exacerbate damage, wear, and subsequent material loss [27]. Because biologic responses to wear debris are influenced by both the amount of debris and the particle characteristics (i.e., size, shape, material), the benefit of using VE-HXLPE in the current TJR design is two-fold; not only is VE-HXLPE associated with significantly less wear than conventional UHMWPE, the wear particles have also been associated with reduced inflammatory potential [13,46,47].

The current study represents one of the earliest investigations into lumbar TJR and may thus serve as a foundation for future work. Potential research stemming from our study includes wear particle analysis and finite element modeling (in particular for impingement scenarios). Already, our encouraging in vitro findings from wear and impingement testing have supported initiating an FDA-regulated clinical trial for the design, which is currently under way [48]. Future work including the evaluation of patient outcomes and clinical performance are expected. Eventual retrieval analyses may also be informed by our current study.

## 5. Conclusions

In summary, the results of this study demonstrated low wear rates for a VE-HXLPE-on-CoCr lumbar total joint replacement. The adverse testing results support that the VE-HXLPE is resistant to abrasive damage and is reasonably forgiving under high stress impingement conditions and is thus suitable for use in this particular spinal application. The potential clinical benefits of this novel design, which avoids long-term facet complications through facet removal with a posterior approach, were found to be effectively balanced by the in vitro tribological performance of the VE-HXLPE bearings.

## Figures and Tables

**Table 1:** Summary of VE-HXLPE lumbar TJR wear rates from the present study in comparison to ADR designs (all incorporating conventional UHMWPE), based on the previous public summaries of safety and effectiveness required by the FDA.

Test	VE-HXLPE TJR	activL SSED [34]	ProDisc SSED [31]
Standard Wear Testing	The average mass wear rate up to 10 MC was 1.2 ± 0.5 mg/MC for the superior UHMWPE components and 0.4 ± 0.1 mg/MC for the inferior (CoCr) components.	Average cumulative wear at 10 million cycles was 25.3 mg and the mean wear rate was 2.7 mg/MC. The test setup was unable to create any backside wear of the polyethylene inlay.	The average mass wear rate of the polyethylene insert up to 5.0 MC was 5.4 ± 1.3 mg/MC and 4.8 ± 1.1 mg/MC for the large- and medium-sized devices, respectively.
Adverse Abrasive Testing	The average mass wear rate up to 5 MC was 1.1 ± 0.6 mg/MC for the superior UHMWPE components and 0.2 ± 0.1 mg/MC for the inferior (CoCr) components.	Not included in SSED.	Not included in SSED.
Impingement Testing	The average mass wear rate up to 1.0 MC was 1.7 ± 1.1 mg/MC (short) and 3.9 ± 1.1 mg/MC (long) for the superior UHMWPE components and 1.2 ± 0.4 mg/MC (short) and 1.2 ± 0.1 mg/MC (long) for the inferior (CoCr) components.	Impingement behavior of the activL^®^ included contact between the cobalt chromium endplates. Based on gravimetric measurements, the mean total material loss from both endplates was 1.5 ± 0.4 mm^3^. The UHMWPE inlays gained mass during testing.Volume to mass loss using 8.4 mg/mm^3^ for density: 12.6 ± 3.4 mg/MC.	Under impingement conditions, the rate of mass loss of the polyethylene insert was less than in the Mode I testing condition. The polyethylene demonstrated impingement on the posterior surface. Characteristic of the contact observed on published retrievals, metal-on-metal contact was observed in the 2 mm offset test group. The maximum mass loss experienced by the metal components was converted to maximum volume losses of 0.6 mm^3^ and 0.5 mm^3^ for the inferior and superior components, respectively.Volumes to mass loss using 8.4 mg/mm^3^ for density: 5.4 mg/MC.

SSED = Summary of Safety and Effectiveness Data, “a document mandated by the Food, Drug and Cosmetic Act subparagraph 520(h)(1)(A) to be publicly available upon issuance of an approval order of a premarket approval application (PMA)” [35].

**Table 2:** Summary of previously reported clean standard wear studies of lumbar ADRs from the literature. These studies all evaluated CoCr-on-conventional UHMWPE lumbar ADR designs.

Authors	Lumbar ADR Design Evaluated	Clean Standard Wear Test Methods	Wear Rate	Notes
Hyde et al. (2017) [36]	CHARITÉ	ISO 18192-1 for 5 MC (“Baseline”), followed by testing a lower cross shear, lower loads, and changes in center of rotation	14.4 ± 2.1 mm^3^/MC	Lower cross shear reduced baseline wear by 49%
Siskey et al. (2016) [27]	CHARITÉ	ISO 18192-1	13.8 ± 3.8 mg/MC	Conventional polyethylene cores were reverse engineered and tested with retrieved endplates
Vicars et al. (2012) [37]	CHARITÉ	ISO 18192-1 for 5 MC (“4DOF”), then 5 MC with additional shear load profile (“5DOF”)	12.2 ± 1.0 mg/MC for 5 MC standard test (“4DOF”); 22.3 ± 2.0 mg/MC for 5 MC standard test (“5DOF”);	Height loss of polyethylene cores was not sensitive to changes in the standard test method
Kettler et al. (2012) [38]	ProDisc L	ISO 18192-1 for 6 MC at 1 and 2 Hz	5.6 ± 2.3 mg/MC at 1 Hz 7.7 ± 1.6 mg/MC at 2 Hz	Authors recommended testing at 1 Hz
Grupp et al. (2009) [39]	Activ-L	ISO/FDIS18192-1 (2006) for 10 MC followed by ASTM F2423-05	2.7 ± 0.3 mg/MC (ISO test method) 0.14 ± 0.06 mg/MC (ASTM 2005 test method)	ASTM 2005 lower wear rate explained by linear wear track
Serhan et al. (2006) [40]	CHARITÉ	Adaptation of ASTM Draft 2 Protocol	0.13 mg/MC	Linear wear track explains low wear rate

**Table 3:** Summary of previously reported impingement studies of anterior lumbar disc replacement designs from the literature. These studies all evaluated CoCr-on-conventional UHMWPE lumbar ADR designs.

Authors	Lumbar TDR Design Evaluated	Test Methods	Wear Rate	Notes
Siskey et al. (2016) [27]	CHARITÉ	Impingement protocols (1 MC) with and without facet engagement developed	−1.0 ± 1.2 mg/MC (wear not detectable due to UHMWPE deformation and mass gain due to fluid adsorption)	Test protocols and impingement scars validated with clinical retrievals
Grupp et al. (2015) [41]	Activ-L	Four different protocols: flexion, extension, lateral bending, flexion and bending	Flexion: 0.67 mm^3^/MC Extension: 0.21 mm^3^/MC Lateral bending: 0.06 mm^3^/MC Flexion and bending: 1.44 mm^3^/MC	CoCr-CoCr endplate impingement simulated, validated with clinical retrievals

## Data Availability

The data presented in this study are available on request from the corresponding author.
